# Photovoltaic Array Fault Diagnosis and Localization Method Based on Modulated Photocurrent and Machine Learning

**DOI:** 10.3390/s25010136

**Published:** 2024-12-29

**Authors:** Yebo Tao, Tingting Yu, Jiayi Yang

**Affiliations:** 1 College of Intelligent Manufacturing, Jiaxing Vocational & Technical College, Jiaxing 314036, China; 2 College of Aerospace Science and Technology, Xidian University, Xi’an 710071, China; ttyu@stu.xidian.edu.cn; 3 College of Computer Science and Technology, Xi’an University of Science and Technology, Xi’an 710054, China; 4 Intelligent Equipment Industrial Research Institute, Hai’an & Taiyuan University of Technology Advanced Manufacturing, Hai’an 226602, China

**Keywords:** fault diagnosis, fault location, photovoltaic power systems, solar energy, artificial intelligence, machine learning

## Abstract

Photovoltaic arrays are exposed to outdoor conditions year-round, leading to degradation, cracks, open circuits, and other faults. Hence, the establishment of an effective fault diagnosis system for photovoltaic arrays is of paramount importance. However, existing fault diagnosis methods often trade off between high accuracy and localization. To address this concern, this paper proposes a fault identification and localization approach for photovoltaic arrays based on modulated photocurrent and machine learning. By irradiating different frequency-modulated light, this method separates photocurrent and directly measures the photoelectric conversion efficiency of each panel, achieving both high accuracy and localization. Through machine learning classification algorithms, the current amplitude and frequency of each photovoltaic panel are identified to achieve fault identification and localization. Compared to other methods, the strengths of this method lie in its ability to achieve high-speed and high-accuracy fault identification and localization by measuring only the short-circuit current. Additionally, the equipment cost is low. The feasibility of the proposed method is demonstrated through practical experimentation. It is determined that when utilizing a neural network algorithm, the fault identification speed meets measurement requirements (5800 obs/s), and the fault diagnosis accuracy is optimal (97.8%).

## 1. Introduction

Photovoltaic energy is one of the cleanest and most available renewable resources, which has attracted much attention in recent decades. The global photovoltaic power generation capacity is growing rapidly. Data show that solar PV will reach 4.7 terawatts (4674 GW) by 2050 [1]. Photovoltaic arrays are often exposed to outdoor conditions throughout the year, subject to diverse environmental influences, and susceptible to degradation, cracks, open circuits, and other faults. These issues result in reduced power generation and even pose a risk of fires. Hence, the establishment of an effective fault diagnosis system for photovoltaic arrays is of paramount importance.

The existing photovoltaic array fault diagnosis methods mainly include the physical characteristics detection method, energy loss detection method, I-V curve detection method, and sequential voltage and current detection method. 1. The physical characteristics detection method analyzes the physical characteristics of the fault module to realize fault detection through the infrared image generated by electroluminescence [2,3], thermal imaging [4,5,6], ultrasonic [7] and other information. Fault diagnosis equipment based on electroluminescence captures the electroluminescence images of photovoltaic panels using a high-resolution infrared camera. Fault areas are identified using image recognition algorithms. Equipment based on thermal imaging determines fault areas by analyzing the temperature distribution of photovoltaic panels during operation. Ultrasonic-based diagnostic equipment locates and identifies crack positions by assessing the impact of cracks on sound wave propagation in photovoltaic panels. These methods can locate faults but struggle with identification and require expensive equipment. Cost estimates for the above-mentioned equipment are presented in Table S1. 2. The energy loss detection method estimates the theoretical output voltage, current, and power by measuring the ambient temperature and irradiance, and then calculates the difference between the theoretical value and the actual value. The difference is used as the input of the diagnosis algorithm to realize fault diagnosis [8,9,10,11]. This method can carry out continuous fault diagnosis and identification when the photovoltaic array is working, but the diagnosis accuracy depends on the accuracy of the simulation model and cannot locate the fault. 3. The diagnosis method based on I-V curve uses the characteristic information contained in the I-V curve of the photovoltaic array to realize fault diagnosis [12,13,14]. This method accurately diagnoses faults by measuring the output characteristics of photovoltaic arrays but lacks localization capability. 4. The sequential voltage and current detection method carries out fault identification by online measuring the output waveform of photovoltaic array [15,16,17,18]. This method can diagnose and identify the fault when the photovoltaic array is working, and it does not need to measure the illumination irradiance and temperature, but it cannot locate the fault. In summary, current methods cannot simultaneously identify, classify, and locate faults with high accuracy, and those with localization capability are costly and less accurate.

To solve the above problems, this paper proposes a photovoltaic array fault identification and location method based on modulated photocurrent and machine learning. Traditional methods based on the I-V curve can accurately identify the types of faults in photovoltaic arrays but cannot localize the fault positions. The proposed method combines I-V curve-based diagnosis with frequency division multiplexing (FDM). This method combines high diagnostic accuracy with fault localization capability. Additionally, leveraging the powerful learning capabilities of machine learning models, this method offers stronger resistance to environmental noise compared to traditional I-V curve-based methods. Environmental noise affects measurement results in complex ways. Machine learning models can mitigate these effects by learning patterns and relationships within the data. In this method, each photovoltaic panel is irradiated with optical signals with different modulation frequencies to generate photocurrent with different frequencies. The short-circuit current amplitude of each frequency (that is, the short-circuit current amplitude of each panel) is obtained by a fast Fourier transform. Finally, the machine learning classification algorithm is used to identify the amplitude and frequency of short-circuit current to realize fault identification and location. To prove the feasibility of this method and compare the recognition effect of seven machine learning algorithms, an experimental platform is built for training and testing. The results show that this method can realize the functions of photovoltaic array fault identification, classification and location simultaneously and has high identification accuracy and low equipment cost. The cost estimates for different diagnostic equipment with fault location capability are detailed in Table S1.

## 2. Principle of Fault Identification and Location for Photovoltaic Array

### 2.1. Theory Analysis

In this paper, a photovoltaic array fault identification and location method based on modulated photocurrent and machine learning is proposed. Firstly, each photovoltaic panel in the array is irradiated with modulated light of different frequencies, and then the short-circuit current spectrum is measured. Finally, the amplitude and frequency of the current spectrum are identified by a machine learning classification algorithm to realize fault identification and location. Short-circuit current as the sole diagnostic parameter provides several advantages: 1. Cost Reduction: It minimizes the number of measurement devices or sensors, thereby lowering equipment costs. 2. Strong Fault Indication: Short-circuit current directly reflects the panel’s state. Faults cause significant changes in short-circuit current, making it a reliable indicator. 3. Reduced Noise Interference: Compared with the multi-parameter methods, single-parameter can reduce the measurement noise. This enhances the stability and reliability of fault diagnosis. 4. Simpler and More Efficient Model: Multi-parameter methods require more complex data set and model structure. Using short-circuit current as the sole diagnostic parameter reduces data preprocessing complexity and improves model efficiency.

**Figure 1:**
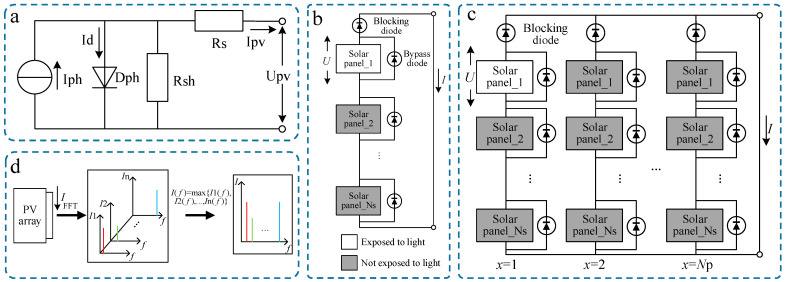
The photovoltaic array models. (**a**) Equivalent circuit of photovoltaic panel. (**b**) Schematic diagram of series photovoltaic array. (**c**) Schematic diagram of series-parallel photovoltaic array. (**d**) Schematic diagram of current spectrum generation.

The circuit model of a single photovoltaic panel can be composed of a diode (D_ph_) and a current source (*I*_ph_) in parallel. The equivalent circuit is shown in Figure 1a. *I*_ph_ is used to represent the photogenerated current characteristics of photovoltaic panels, and its amplitude is proportional to the irradiance. D_ph_ is used to represent the nonlinear output characteristics of photovoltaic panels. Equivalent series resistance (*R*_s_) and equivalent parallel resistance (*R*_sh_) are used to represent the losses caused by defects in substrate materials, metal wires and contact points. The equivalent circuit mathematical model for photovoltaic panels with different power ratings can be expressed by (1):

(1) $$I_{\mathrm{pv}}=I_{\mathrm{ph}}-I_{\mathrm{rs}}\left[\exp\left(\frac{q\left(U_{\mathrm{pv}}+IR_{s}\right)}{AkT}\right)-1\right]-\frac{U_{\mathrm{pv}}+IR_{s}}{R_{\mathrm{sh}}}$$

where *I*_pv_ and *U*_pv_ are the output current and output voltage of the photovoltaic panel, in units of A and V, *I*_ph_ is the photocurrent, in units of A, *q* is the amount of electronic charge, *k* is the Boltzmann constant, *A* is the diode characteristic factor, *T* is the absolute temperature of the battery, in units of K, *I*_rs_ is the diode reverse saturation current, in units of A, *R*_s_ is the equivalent series resistance, *R*_sh_ is the equivalent parallel resistance, in units of Ω. Generally, *R*_sh_ is much larger than *R*_s_, and *R*_s_ ≈ 0; then (1) can be rewritten as (2). The analysis and simulation of the impact of *R*_s_ and *R*_sh_ on the measurement error of the short-circuit current can be found in Supplementary Note S1.

(2) $$I_{\mathrm{pv}}=I_{\mathrm{ph}}-I_{\mathrm{rs}}\left[\exp\left(\frac{qU_{\mathrm{pv}}}{AkT}\right)-1\right]$$

To meet power demands, photovoltaic panels are connected in series and parallel to form arrays. Photovoltaic arrays commonly use series and series-parallel connections.

#### 2.1.1. Series Connection

Series connection means that each photovoltaic panel is connected in series to form a photovoltaic array, which has the characteristics of high output voltage. The circuit schematic is shown in Figure 1b. The bypass diodes (D_s1_–D_sn_) are used to avoid the occurrence of hot spot [19,20]. The blocking diode (D_s0_) prevents reverse current flow [21]. Photovoltaic arrays exhibit a variety of fault types, including degradation, open-circuit, partial shading conditions, hot spots, and more. Partial shading conditions due to bird dropping and dust accumulation can be easily identified by appearance. Photovoltaic arrays typically use bypass diodes to avoid the occurrence of hot spot [19,20]. Open-circuit and degradation faults are the most common in photovoltaic arrays [18,22]. Therefore, this paper focuses on these two specific fault states. Moving forward, we will further research other fault states.

When the modulated light irradiates the photovoltaic array, it is assumed that there is one LED above the photovoltaic panel at time *t*, the total number of photovoltaic panels in series is *N*_s_, and the output of the photovoltaic array is short-circuited. At this time, the output voltage of the photovoltaic panel exposed to light is *U*, and the bypass diodes connected in parallel to the photovoltaic panels not exposed to light are conducting. The conducting bypass diodes and the blocking diode together serve as the load for the photovoltaic panel exposed to light. Then the output current I can be expressed as [23,24,25]:

(3) $$I=I_{\mathrm{ph}}\left(t\right)-I_{\mathrm{rs}}\left(\exp\left(\frac{qU}{AkT}\right)-1\right)$$

The output voltage *U* can be expressed as:

(4) $$U=U_{d}N_{s}$$

where *U*_d_ refers to the forward voltage drop of the bypass diode and blocking diode.

From (3) and (4), it can be concluded that in the series connection, the modulated optical signal will eventually make the short-circuit current include the alternating component *I*_n_(*t*) through *I*_ph_(*t*). The modulated optical signal enables the photovoltaic panel to switch between two states, one in the absence of light and the other in the presence of light at a specific frequency (equal to the frequency of the modulated optical signal). When exposed to light, the conducting bypass diodes and the blocking diode together serve as the load for the photovoltaic panel. The photovoltaic panel outputs a corresponding current based on different conditions in this state. In the normal state, the output current value is maximum. In the presence of a fault, the output current value decreases to varying degrees depending on the fault state. When not exposed to light, there is no output current. The impact of the modulated optical signal on current amplitude for different fault states is shown in Figure S1. Therefore, the fault type can be determined by amplitude, and the position of the faulty photovoltaic panel can be determined by frequency.

The above analysis is based on the condition that only one LED above the photovoltaic panel emits light at the same time. To facilitate the overall analysis of the photovoltaic array, *I*(*f*) is obtained through (5), as shown in Figure 1d, and *I*(*f*) is used for fault diagnosis.

(5) $$I\left(f\right)=\max\left\{I_{1}\left(f\right),I_{2}\left(f\right),\ldots ,I_{n}\left(f\right)\right\}$$

where *I*(*f*) is the current spectrum used for fault diagnosis, *I*_n_(*f*) is the short-circuit current spectrum obtained by each photovoltaic panel separately irradiating modulated light with different frequencies, and *n* is the total number of photovoltaic panels in the photovoltaic array.

#### 2.1.2. Series-Parallel Connection

Series-parallel connection means that each photovoltaic panel is connected in series and then in parallel to form a photovoltaic array, which has the characteristics of high output power. Its schematic diagram is shown in Figure 1c. It is assumed that at time *t*, there is a photovoltaic panel on a series branch illuminating modulated light, the total number of photovoltaic panels in the series of the branch is *N*_s_, and the photovoltaic array output is short-circuited. At this time, the output voltage of the photovoltaic panel exposed to light is *U*, and the bypass diodes connected in parallel to the photovoltaic panels not exposed to light are conducting. The conducting bypass diodes and the blocking diode together serve as the load for the photovoltaic panel exposed to light. *I* and *U* can still be expressed by (3) and (4).

Therefore, the fault type can be determined by amplitude, and the position of the faulty photovoltaic panel can be determined by frequency.

### 2.2. Simulation Verification

#### 2.2.1. Simulation of Series Connection

**Figure 2:**
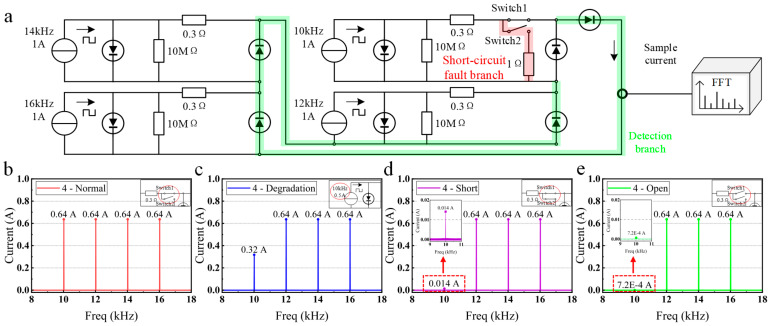
The simulation circuit and results of a series-connected photovoltaic array. (**a**) Series-connected simulation circuit. (**b**) Simulation results under the normal state. (**c**) Simulation results under degradation fault. (**d**) Simulation results under short-circuit fault. (**e**) Simulation results under open-circuit fault.

To verify the above theoretical analysis, the circuit is simulated and analyzed. The simulation circuit is composed of four photovoltaic panels in series, as shown in Figure 2a. Opening switch 1 simulates an open-circuit state, closing switch 2 simulates a short-circuit state, and reducing the amplitude of the current source simulates a degradation state. Square wave current sources (10 kHz, 12 kHz, 14 kHz, and 16 kHz) simulate panels illuminated by modulated light. The reasons for choosing these four frequencies are as follows: 1. the interval between modulation frequencies needs to be large enough. This can ensure that the short-circuit current generated by different photovoltaic panels has good resolution in the frequency domain. 2. the LED driver and signal processing circuits have good performance in this frequency range. 3. the frequency range avoids the interference of power frequency (50/60 Hz). 4. uniform frequency distribution aids machine learning algorithms in recognizing short-circuit current variations during training. This improves the ability to identify fault types and locations. The output current spectrums are shown in Figure 2b–e.

Figure 2b presents the spectrum of the short-circuit current output from a photovoltaic array composed of four series-connected photovoltaic panels under the normal state. In this scenario, the spectrum of short-circuit current exhibits four distinct high-amplitude current components. These current components’ frequencies correspond to the modulation frequencies of the light on each photovoltaic panel, and their amplitudes reflect the magnitude of the photocurrent generated by each modulated light. To simulate a degradation fault in the photovoltaic panel, switch 1 is kept closed, switch 2 is kept open, and the current source is adjusted from 1 A to 0.5 A. In this condition, the short-circuit current spectrum is shown in Figure 2c. In Figure 2c, only three high-amplitude current components are visible. The amplitude of the current component corresponding to the faulty panel decreases from 0.64 A to 0.32 A. Therefore, by recognizing the amplitude of the current components, it’s possible to determine the presence of degradation faults. Moreover, identifying the frequency of the current components helps locate the faults.

If switch 1 and switch 2 are simultaneously closed, short-circuit faults will be simulated. A 1 Ω resistance in series with switch 2 simulates the equivalent resistance of the short-circuit fault branch. The short-circuit current spectrum can be obtained as shown in Figure 2d. In Figure 2d, the photovoltaic panels at frequencies of 12 kHz, 14 kHz, and 16 kHz are in a normal state, with current amplitudes equivalent to the normal state shown in Figure 2b. The photovoltaic panel at 10 kHz frequency is in a short-circuit fault state, and the current amplitude decreases from 0.64 A to 0.014 A compared to the normal state in Figure 2b. The current amplitude is smaller than the degradation fault state in Figure 2c (0.32 A). A short-circuit fault significantly reduces the current amplitude of the corresponding photovoltaic panel, while the current amplitudes of other photovoltaic panels in normal states remain unchanged.

If switches 1 and 2 are simultaneously open, simulating open-circuit faults resulting from photovoltaic panel fractures or wire disconnections, the short-circuit current spectrum can be obtained, as shown in Figure 2e. In Figure 2e, the photovoltaic panels at frequencies of 12 kHz, 14 kHz, and 16 kHz are in a normal state, with current amplitudes equivalent to the normal state shown in Figure 2b. The photovoltaic panel at 10 kHz frequency is in an open-circuit fault state, and the current amplitude decreases from 0.64 A to 0.72 mA compared to the normal state in Figure 2b. The current amplitude is even smaller than the short-circuit fault state in Figure 2d (0.014 A). This is because the fault branch and the detection branch are in parallel in the short-circuit fault state, so a tiny amount of current is still flowing into the detection branch. In the open-circuit fault state, the photovoltaic panel is wholly disconnected from the detection branch, and theoretically, there is no current flowing into the detection branch. Therefore, the current amplitude in an open-circuit fault state is smaller than in a short-circuit fault state. Hence, by analyzing the frequencies and amplitudes in the short-circuit current spectrum, it is possible to identify the type and location of faults in the photovoltaic array. Simulations confirm the method’s effectiveness in diagnosing and locating faults in series-connected photovoltaic arrays.

#### 2.2.2. Simulation of Series-Parallel Connection

**Figure 3:**
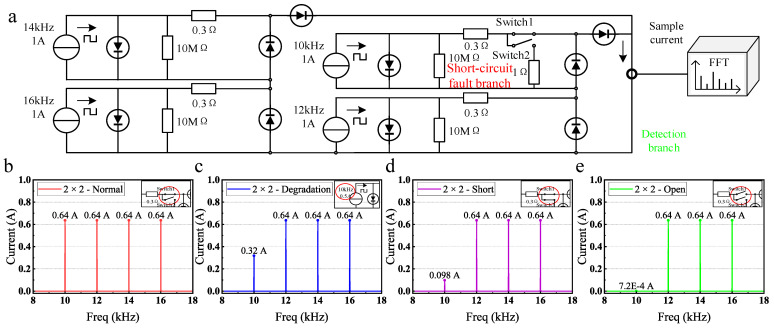
The simulation circuit and results of a series-parallel connected photovoltaic array. (**a**) Series-parallel simulation circuit. (**b**) Simulation results under the normal state. (**c**) Simulation results under degradation fault. (**d**) Simulation results under short-circuit fault. (**e**) Simulation results under open-circuit fault.

The 2 × 2 series-parallel simulation circuit is composed of two photovoltaic panels connected in series and then two of these sets are connected in parallel, as shown in Figure 3a. The simulation methods for different states are the same as those in the simulation of series connection. Four square wave current sources are applied at frequencies of 10 kHz, 12 kHz, 14 kHz, and 16 kHz. The output current spectrums are shown in Figure 3b–e.

The current spectrum in Figure 3b–e is largely consistent with that of Figure 2b–e. This is because, during each measurement process, the load is short-circuited, and only one panel is illuminated. Array structure differences affect only the number of conducting diodes per measurement. Due to the low forward voltage of diodes (approximately 0.7 V), their impact on the output current of the photovoltaic panels is minimal. In Figure 3d, the photovoltaic panel at 10 kHz frequency is in a short-circuit fault state, and the current amplitude (0.098 A) is higher than that in Figure 2d (0.014 A). This is because, under the series-parallel connection, the number of detection branch diodes (2) is less than under the series connection (4). Therefore, the equivalent resistance of the detection branch is minor, and the current distributed in parallel with the short-circuit fault branch is higher. As a result, simulations show the method effectively diagnoses and locates faults in series-parallel arrays.

## 3. Experiments and Results

### 3.1. Experimental Setup

**Figure 4:**
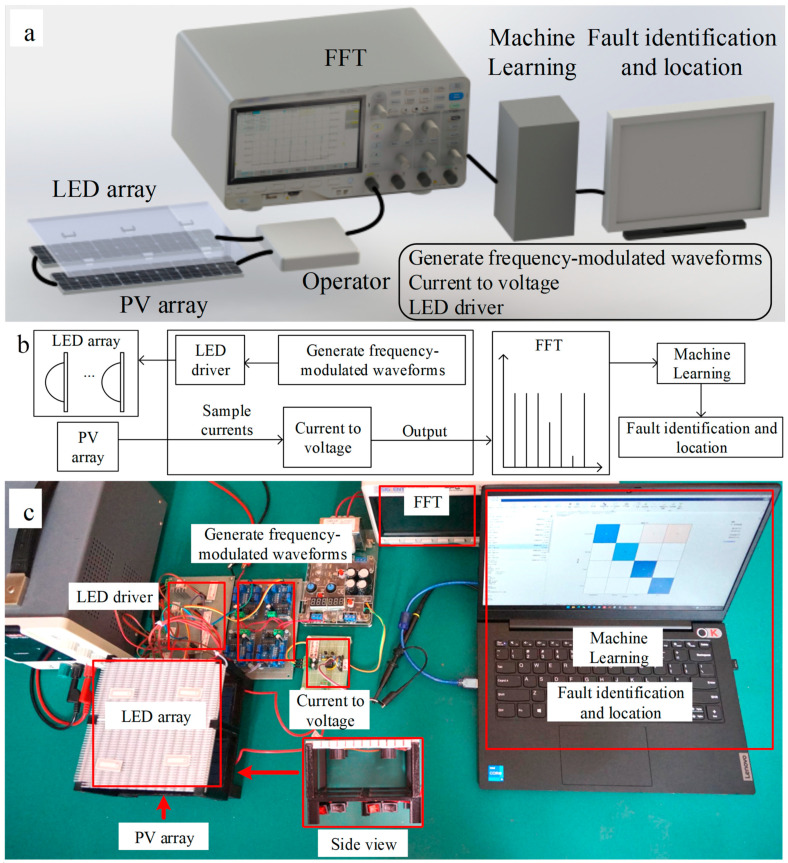
Experimental setup of modulated light generation and current detection. (**a**) Schematic diagram. (**b**) Block diagram. (**c**) Photograph.

A modulated light generation and current detection experimental platform was designed to verify the feasibility of the proposed method. The modulated light generation device was composed of a signal generation module, a voltage-controlled current source module, and an array of LEDs. It can generate a set of specific frequency light sources. This platform utilized the array of LEDs as an actual light source. The three-dimensional schematic diagram and block diagram are shown in Figure 4a,b. The modulated light array emits modulated light at different frequencies, which is irradiated onto each photovoltaic panel of the photovoltaic array. The total short-circuit current is converted to voltage, and the current amplitude at each frequency is obtained through the spectrum analyzer. The photograph of the experimental platform is shown in Figure 4c. The parameters of the experimental platform are presented in Table S2.

**Figure 5:**
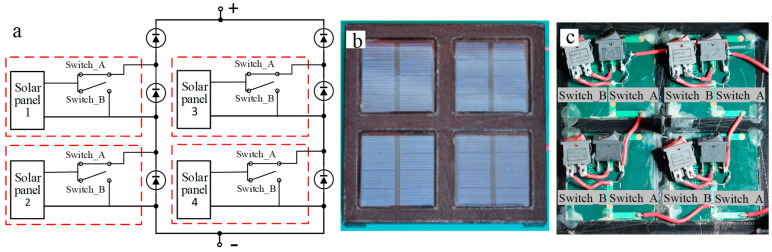
Photovoltaic array capable of switching among normal, open-circuit, short-circuit, and degradation states. (**a**) Circuit schematic diagram. (**b**) Photograph (front view). (**c**) Photograph (back view).

To enable the photovoltaic array to switch among four states: normal, short-circuit, open-circuit, and degradation, each photovoltaic panel’s output is connected to two switches (switch A in series and switch B in parallel). Degradation faults are simulated by placing optical filters, where filters of different sizes simulate varying degrees of degradation. The schematic diagram is shown in Figure 5a. Photographs are shown in Figure 5b,c.

### 3.2. Experiments

**Figure 6:**
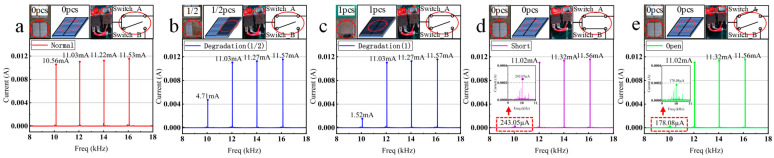
Current spectrum under different states. (**a**) Normal state. (**b**) The photovoltaic panel is irradiated by modulated light at 10 kHz and is covered by half of the optical filter. (**c**) A photovoltaic panel is irradiated by modulated light at 10 kHz and is covered by one optical filter. (**d**) The photovoltaic panel is irradiated by modulated light at 10 kHz in a short-circuit state. (**e**) The photovoltaic panel is irradiated by modulated light at 10 kHz in an open-circuit state.

This paper measured the short-circuit current spectrum of the photovoltaic array under different states using the experimental setup. Ten sets of data were measured for each state. The average value was taken as the final result to ensure data consistency and representativeness. The results are shown in Figure 6a–e.

Figure 6a shows the short-circuit current spectrum of the 2 × 2 serial-parallel photovoltaic array under the normal state. From Figure 6a, it can be observed that there are four current components with distinct high amplitudes. The frequencies of these four current components are equal to the frequencies of the modulated light irradiating the four photovoltaic panels. The amplitude of each current component is approximately equivalent to the amplitude of the photocurrent generated by each modulated light. In Figure 6a, the amplitudes of the four current components are not entirely equal. This discrepancy arises due to three factors: (1) inconsistent photovoltaic panel conversion efficiencies, (2) inconsistent emission intensities from the LED array, and (3) structural errors.

Studies have shown that the degradation of photovoltaic panels significantly affects the short-circuit current. As the degradation level increases, the amplitude of the short-circuit current generated by the photovoltaic panels decreases. However, degradation faults do not lead to a decrease in the short-circuit current to zero [22]. Therefore, this paper employed optical filters to cover the surface of photovoltaic panels in two ways: half coverage and full coverage, simulating different degrees of degradation faults.

To simulate a degradation fault in the photovoltaic panel, switch A was kept closed, switch B was kept open, and half of an optical filter was placed above the photovoltaic panel to simulate degradation. The short-circuit current spectrum is shown in Figure 6b. In Figure 6b, only three current components exhibit distinct high amplitudes. The current component corresponding to the faulty panel shows a decrease in amplitude from 10.56 mA to 4.71 mA.

To simulate severe degradation faults in the photovoltaic panel, an optical filter was used to completely cover the surface of the photovoltaic panel. The resulting short-circuit current spectrum is shown in Figure 6c. In Figure 6c, only three current components exhibit distinct high amplitudes. Compared to the normal state in Figure 6a at a frequency of 10 kHz, the current amplitude decreases from 10.56 mA to 1.52 mA. It is lower than the simulated degradation state (4.71 mA) in Figure 6b with half coverage. This is due to the severe degradation fault simulated by the full coverage, which blocks more light than the degradation fault simulated by the half coverage, causing the further reduction of the photocurrent. The experimental data in Figure 6b,c demonstrate that greater degradation results in a larger decrease in short-circuit current amplitude.

To simulate a short-circuit fault in the photovoltaic panel, switch A and switch B were set to a close position simultaneously. The resulting short-circuit current spectrum is shown in Figure 6d. In Figure 6d, only three current components exhibit distinct high amplitudes. The amplitude of the 10 kHz current component decreases from 10.56 mA to 0.243 mA, which is smaller than the amplitude under the severe degradation fault state (1.52 mA).

Setting switches A and B to open simulates an open-circuit fault. The resulting short-circuit current spectrum is shown in Figure 6e. In Figure 6e, only three current components exhibit distinct high amplitudes. The 10 kHz current amplitude drops from 10.56 mA to 0.178 mA, lower than in the short-circuit state (0.243 mA). However, it does not completely vanish, which is attributed to the space between the LED array and the photovoltaic panel allowing light to be reflected onto the surface of other photovoltaic panels. This error can be reduced through structural improvements.

Therefore, analyzing short-circuit current frequencies and amplitudes enables fault type and location identification. The experimental results demonstrate the effectiveness of this method for diagnosing and locating faults in photovoltaic arrays.

### 3.3. Results and Analysis

Based on the results of simulation and experimentation, it is evident that the short-circuit current generated under modulated light illumination exhibits frequency modulation. This paper diagnoses the fault states of photovoltaic panels based on the amplitude of short-circuit current. However, there is no analytical solution between the fault states and short-circuit current, and the fault states are remarkably diverse. Therefore, this paper employed machine learning algorithms to identify current signals, aiming to accurately diagnose the fault states and locations in the photovoltaic array. This paper compared seven mainstream machine learning algorithms: k-Nearest Neighbors, Discriminant Analysis, Naive Bayes, Decision Trees, Ensemble Learning, Support Vector Machines, and Neural Networks [26,27,28,29,30,31]. Neural networks are a computational model that emulates the human brain’s neural system, with numerous neurons interconnected to learn and simulate complex nonlinear relationships. Neural networks, through multiple input, hidden, and output layers, leverage nonlinear modeling and adaptive learning capabilities, enabling effective capture of complex nonlinear relationships within the photovoltaic array and adaptation to changes in various parameters of photovoltaic panels. The advantages of applying it to diagnosing faults in photovoltaic arrays include nonlinear solid modeling capabilities, excellent feature learning abilities, good generalization capabilities, and outstanding robustness and fault tolerance.

**Figure 7:**
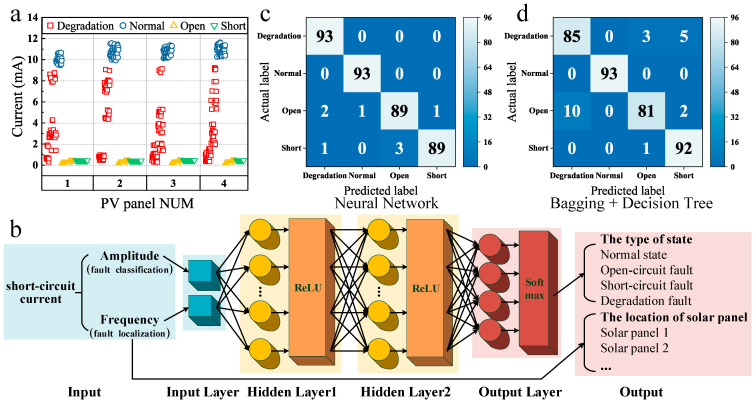
Data set and results. (**a**) Distribution of the data set. (**b**) Neural Network architecture. (**c**) Testing confusion matrix (Neural Network). (**d**) Testing confusion matrix (Ensemble Learning: Bagging + Decision Tree).

To improve generalization, the data set was collected in indoor and outdoor conditions. Temperatures ranged from 10 °C to 30 °C, and illumination levels ranged from 100 lux to 10,000 lux. Seventy percent of the data was used for training, and the remaining 30% for testing. This paper used various classifiers for training and optimized parameters through cross-validation. Finally, the model was utilized to recognize the test set and obtain the accuracy of the test set. Based on the test accuracy and prediction speed, a final assessment was conducted to determine the optimal recognition algorithm and model. Experimental results are shown in Figure 7 and Table 1.

To obtain the data set for model training, this paper configured the photovoltaic array in various states (1. normal, 2. short-circuit fault, 3. open-circuit fault, and 4. degradation fault), resulting in a collection of 1240 data sets. The detailed process of generating and collecting the data set can be found in Supplementary Note S3. The data distribution is shown in Figure 7a, where “Normal” means the normal state, “Open” means the open-circuit fault, “Short” means the short-circuit fault, and “Degradation” means the degradation fault. From Figure 7a, it is apparent that the distribution of current amplitudes under different states overlaps, making it impossible to differentiate between the states using specific threshold values. Hence, it is necessary to utilize machine learning classification algorithms for fault diagnosis and localization.

From Table 1, it can be observed that all seven selected machine learning algorithms can diagnose faults in the photovoltaic array based on current amplitude and frequency, and their prediction speeds meet measurement requirements. The Neural Network achieved the highest testing accuracy (97.8%), attributed to its strong capacity for nonlinear modeling, robustness, and tolerance to noisy data, as well as its good generalization ability to unseen data. The Ensemble Learning (Bagging + Decision Tree, i.e., Random Forest) demonstrated a relatively high test accuracy (94.4%), owing to its combination of predictions from multiple decision tree classifiers. Each decision tree is constructed based on randomly selected feature subsets and sample subsets, leading to reduced variance, the ability to mitigate overfitting issues, and enhanced overall classification accuracy.

Figure 7b illustrates the neural network structure, consisting of one input layer, two hidden layers, and one output layer. Bayesian optimization is employed for neural network hyperparameter tuning to obtain optimal hyperparameters. The hyperparameter optimization process of the neural network is detailed in Figure S5. Each hidden layer consists of 100 neurons, and the Rectified Linear Unit (ReLU) is used as the activation function for each hidden layer. The output layer employs the Softmax activation function for classifying fault types in the photovoltaic array. During training, the cross-entropy loss function is utilized. To prevent model overfitting, this paper employs the early stopping strategy to halt training as soon as the model’s performance on the validation set ceased to improve. Additionally, L2 regularization is introduced to limit the magnitude of model parameters, and dropout layers are used to randomly drop neurons, enhancing the model’s generalization. The current frequency is employed to determine the location of the photovoltaic panel. Figure 7c,d show the testing confusion matrices for the Neural Network and the Ensemble Learning, respectively. The results indicate that the Neural Network achieved the highest accuracy, albeit with a slightly slower recognition speed compared to other machine learning algorithms, still meeting measurement requirements. Therefore, this paper selected the Neural Network as the photovoltaic array fault diagnosis algorithm. Based on the Neural Network, the proposed photovoltaic array fault diagnosis and localization method based on modulated photocurrent and machine learning achieved an accuracy of 97.8%. To verify the applicability of the method proposed in this paper, we conducted experiments using a more complex series-parallel photovoltaic array structure, as detailed in Supplementary Note S3.

Table 2 compares the method presented in this paper with other fault diagnosis methods for photovoltaic arrays across five dimensions: the ability to locate faults, the number of measured parameters, accuracy, recognition speed and equipment cost. The comparison shows that the innovation of the proposed method lies in the significant benefits brought about by integrating the fault diagnosis method based on the I-V curve with frequency division multiplexing (FDM) technology. The proposed method requires measuring only a single parameter: the short-circuit current. It enables high-speed and high-accuracy fault detection through current amplitude. It also allows fault localization through current frequency. The method proposed in this paper offers advantages in terms of detection accuracy, localization capability, equipment cost, detection speed, and the simplicity of required measurement parameters. A detailed comparison and application analysis of the proposed method and existing methods can be found in Supplementary Note S4.

A demo system was developed to demonstrate the feasibility of the method proposed in this paper, as detailed in Supplementary Note S2 and Video S1. The system comprises an array of LEDs, an LED driver, an I-V conversion circuit, an ARM system, and host software. It is deploying the fault diagnosis model based on neural networks on ARM instead of high-performance computers. This proves the low-cost nature of the proposed approach. This device enables rapid (less than 1 s) diagnosis and localization of four states of the photovoltaic array (normal, short-circuit, open-circuit, degradation), with measurement results unaffected by external light sources. Compared to other methods listed in Table 2, the approach presented in this paper offers high accuracy, fast speed, low cost, minimal measurement requirements, and fault localization capabilities.

## 4. Conclusions

This paper proposes a photovoltaic array fault identification and location method based on modulated photocurrent and machine learning. It addresses the shortcomings of existing detection methods that cannot simultaneously provide fault identification, classification, and localization capabilities, as well as the issues of low accuracy and high equipment costs in detection methods with localization functionality. The paper analyzes the principle of using modulated light to achieve photovoltaic array fault identification and localization and validates it through simulation. Additionally, to demonstrate the feasibility of this method and compare the recognition performance of seven machine learning algorithms, an experimental platform is set up for training and testing. The experimental results show that this method can achieve fault identification and localization in photovoltaic arrays. When using a neural network as the fault diagnosis algorithm, the fault identification speed meets measurement requirements, and the fault diagnosis accuracy is the highest.

Future research will focus on applying the method to larger gigawatt-scale photovoltaic arrays. As the array size increases, fault diagnosis becomes more complex. To improve accuracy and adaptability, we will explore advanced machine learning models (e.g., residual networks, graph neural networks) and novel detection modules (e.g., time series analysis). The complexity of frequency selection will also grow, potentially causing signal interference. To address this, we will investigate advanced signal processing techniques, such as adaptive frequency allocation and filtering, to ensure reliable results. Additionally, the LED array will be improved to accommodate larger photovoltaic panels, and the system structure will be optimized to simplify installation and maintenance.

## Figures and Tables

**Table 1:** Performance Comparison of Different Classifier Algorithms.

Model	Hyperparameters	Prediction Speed	Accuracy (Validation)	Accuracy (Test)
k-Nearest Neighbors	Number of Neighbors: 10 Distance Metric: Euclidean Distance Weight: Equidistant	10,000 obs/s	96.0%	85.9%
Discriminant Analysis	Discriminant Type: Quadratic	17,000 obs/s	93.1%	90.2%
Naive Bayes	Distribution Name: Kernel Kernel Type: Box	10,000 obs/s	95.2%	92.4%
Decision Trees	Maximum Number of Splits: 14 Split Criterion: Gini’s diversity index	20,000 obs/s	97.6%	89.1%
Ensemble Learning	Ensemble Method: Bag Maximum Number of Splits: 22 Learner Type: Decision tree Number of Learners: 126	1100 obs/s	98.2%	94.4%
Ensemble Learning	Ensemble Method: RUSBoost Maximum Number of Splits: 22 Learner Type: Decision tree Number of Learners: 213	25,000 obs/s	97.8%	95.2%
Ensemble Learning	Ensemble Method: AdaBoost Maximum Number of Splits: 13 Learner Type: Decision tree Number of Learners: 43	5300 obs/s	97.1%	94.5%
Support Vector Machines	Kernel Function: Cubic Box Constraint Level: 1 Multiclass Method: One-vs-One	13,000 obs/s	97.3%	91.3%
Neural Networks	Number of Fully Connected Layers: 2 Layer Size: 100 Activation: ReLU, Softmax Regularization Strength (Lambda): 0	5800 obs/s	98.7%	97.8%

Note: “obs/s” represents observations per second.

**Table 2:** Comparison of Photovoltaic Array Fault Diagnosis Methods.

Ref.	Detection Method	The Ability to Locate Faults	Online Detection	The Number of Measured Parameters	Accuracy	Recognition Speed	Equipment Costs
this work	Proposed detection method	Yes	No	1 (Isc)	97.8%	<1 s	Low
[2,3,4,5,6,7]	Physical characteristics detection method	Yes	Electroluminescence imaging and ultrasonic wave: No Thermal imaging: Yes	1 (electroluminescence imaging or thermal imaging or ultrasonic wave)	Indirect measurements	25–120 s	Very high
[8,9,10,11]	Energy loss detection method	No	Yes	4 (irradiation, temperature, Upv, Ipv)	95.3–98.8%	1–5 min	High
[12,13,14]	I-V curve detection method	No	No	2 (Upv, Ipv)	95.8–99.7%	<1 s	Low
[15,16,17,18]	Sequential voltage and current detection method	No	Yes	2 (Upv, Ipv)	91.4–94.7%	<1 s	Low

## Data Availability

The data presented in this study are available upon request from the corresponding author.

## Supplementary Materials

The following supporting information can be downloaded at: https://www.mdpi.com/article/10.3390/s25010136/s1, Figure S1: The impact of the modulated optical signal on current amplitude for different fault states; Figure S2: The impact of simplifying (1) with (2) on the method presented in this paper; Figure S3: The demo system based on the method in this paper, shown in the video (Supplementary Video S1); Figure S4: How to generate and collect the data; Figure S5: The minimum classification error; Figure S6: The 3 × 3 series-parallel photovoltaic array and experimental setup; Figure S7: Data set and experimental results of a 3 × 3 series-parallel photovoltaic array; Figure S8: The application and comparison of online and offline fault detection methods for photovoltaic arrays; Figure S9: Using the method proposed in this paper to perform automated fault detection and localization on photovoltaic arrays during the production process; Figure S10: Using the method proposed in this paper to perform automated fault detection and localization on outdoor-installed photovoltaic arrays during the maintenance process; Table S1: The Cost Estimates For Different Diagnostic Equipment With Fault Location Capability; Table S2: The Parameters of The Experimental Platform; Video S1: demo system; Supplementary Note S1: The analysis of the impact of simplifying (1) with (2) on the method presented in this paper; Supplementary Note S2: A demo system for fault diagnosis of photovoltaic panel arrays based on the method proposed in this paper; Supplementary Note S3: A more complex series-parallel photovoltaic array structure is used to verify the feasibility and performance of the method in this paper; Supplementary Note S4: A detailed comparison and application analysis of the proposed method and existing methods. References [32,33,34] are cited in the Supplementary Materials.
