# Supplementary material for: Photovoltaic Array Fault Diagnosis and Localization Method Based on Modulated Photocurrent and Machine Learning
Source: Sensors (Basel). 2024 Dec 29;25(1):136. doi: 10.3390/s25010136 (PMC11723431; doi:10.3390/s25010136)
Supplement: Supplementary file 1 [file sensors-25-00136-s001.zip › sensors-3387730-supplementary.pdf]

Supplementary Information

Photovoltaic Array Fault Diagnosis and Localization Method Based on Modulated Photocurrent and Machine Learning

Yebo Tao [1,*], Tingting Yu [2] and Jiayi Yang [3,4,*]

1 College of Intelligent Manufacturing, Jiaxing Vocational & Technical College, Jiaxing 314036, China

2 College of Aerospace Science and Technology, Xidian University, Xi’an 710071, China; ttyu@stu.xidian.edu.cn

3 College of Computer Science and Technology, Xi’an University of Science and Technology, Xi’an 710054, China

4 Intelligent Equipment Industrial Research Institute, Hai’an & Taiyuan University of Technology Advanced Manufacturing, Hai’an 226602, China

***** Correspondence: taoyebo@jxvtc.edu.cn (Y.T.); jyang46@xust.edu.cn (J.Y.)

Supplementary Figures

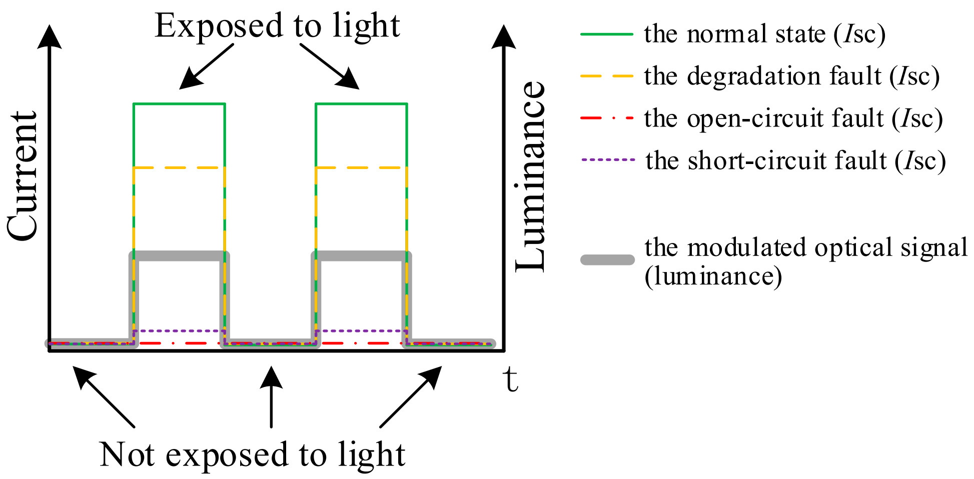

Supplementary Figure S1. The impact of the modulated optical signal on current amplitude for different fault states. The modulated optical signal enables the photovoltaic panel to switch between two states; one in the absence of exposure to light and the other in the presence of exposure to light at a specific frequency (equal to the frequency of the modulated optical signal). When exposed to light, the conducting bypass diodes and the blocking diode together serve as the load for the photovoltaic panel. In this state, the photovoltaic panel outputs a corresponding current based on different conditions. In the normal state, the output current value is maximum. In the presence of a fault, the output current value decreases to varying degrees depending on the fault state. When not exposed to light, there is no output current.

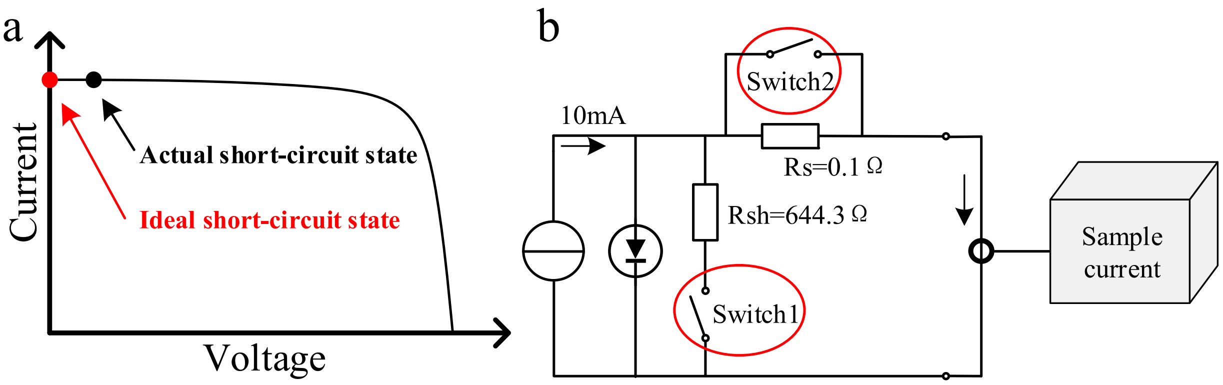

Supplementary Figure S2. The impact of simplifying (1) with (2) on the method presented in this paper. (a) The I-V curve of the photovoltaic panel, with the ideal short-circuit state indicated by the arrow. The equivalent series resistance (R_s) affects the voltage when the photovoltaic panel is short-circuited, causing the corresponding position on the I-V curve to shift to the right to the actual short-circuit state. (b) The simulation circuit, where switch1 and switch2 can control whether R_s and R_{sh} are connected.

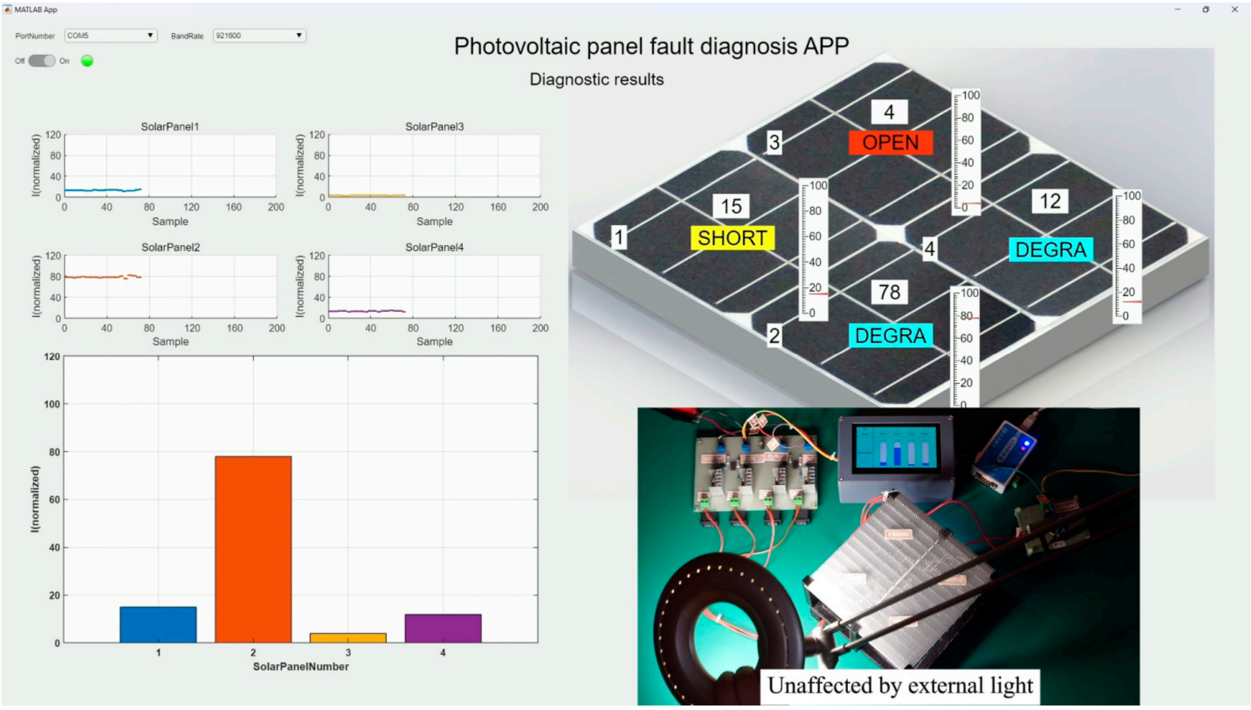

Supplementary Figure S3. The demo system based on the method in this paper, shown in the video (Supplementary Video-1).

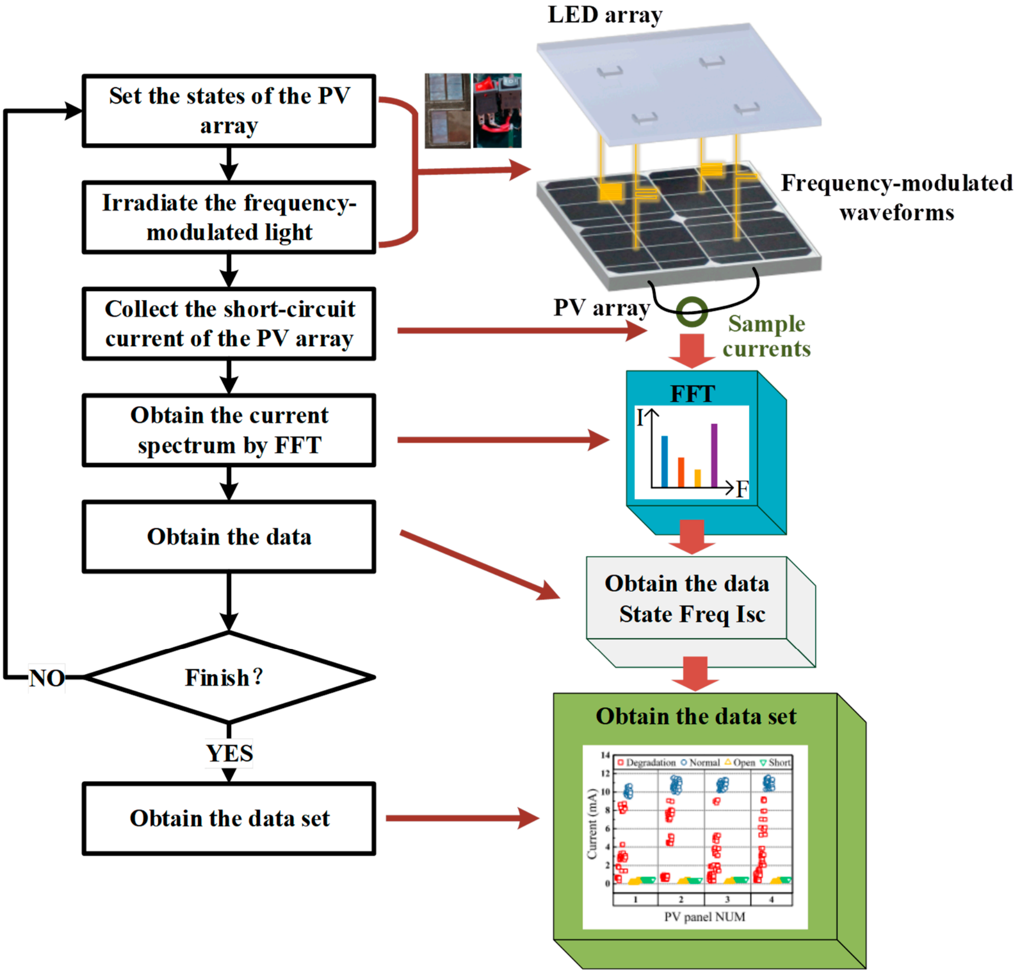

Supplementary Figure S4. How to generate and collect the data. The steps for generating and collecting the short-circuit output current data set of the photovoltaic array under the four states described in this paper are as follows:

1) Set the states of the PV array using switches or an optical filter;

2) Irradiate the frequency-modulated light;

3) Collect the short-circuit current of the PV array;

4) Obtain the current spectrum by FFT;

5) Obtain the data on the states of each PV panel, frequency, and current amplitude at corresponding frequencies;

6) Obtain the data set.

The steps for generating and collecting the data set are shown in the figure.

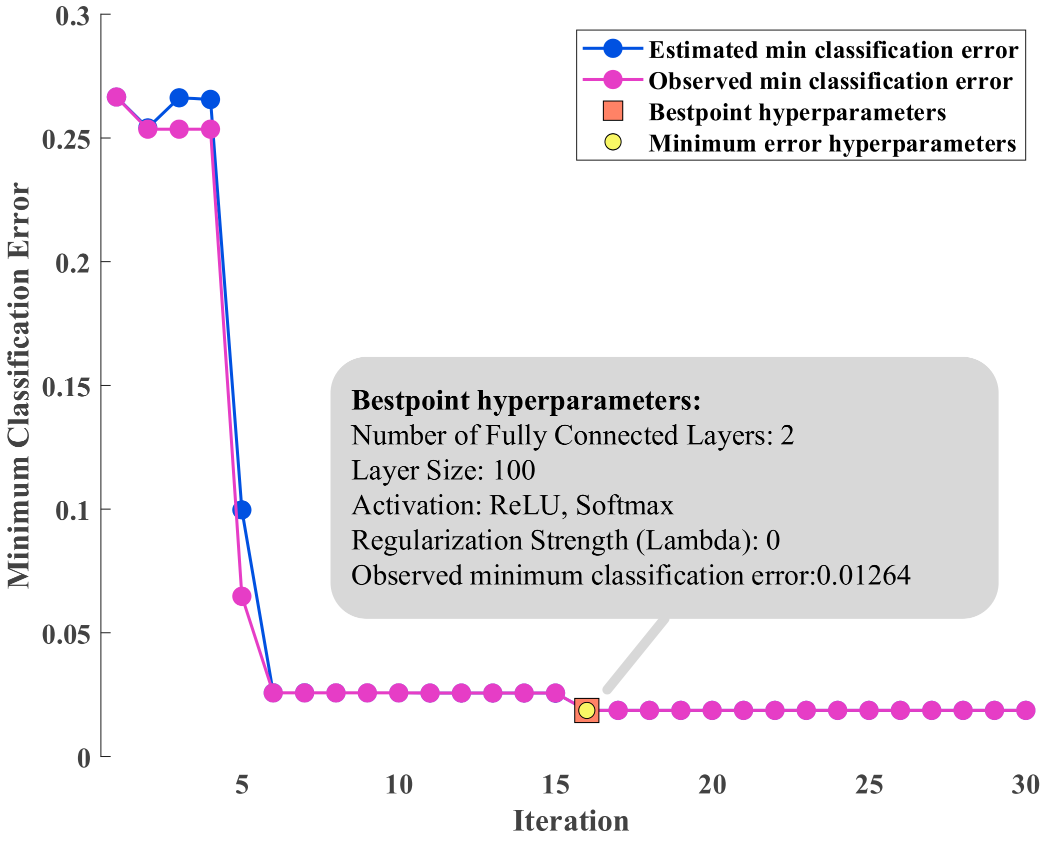

Supplementary Figure S5. The minimum classification error. We employed a Bayesian optimization method for hyperparameter tuning to determine the most appropriate neural network structure. The optimization algorithm reached its minimum classification error and upper confidence interval of classification error by the 16th iteration, resulting in the optimal structure as follows: two hidden layers, each with a size of 100, using the ReLU activation function for the hidden layers and the Softmax activation function for the output layer. The optimization process is shown in the figure.

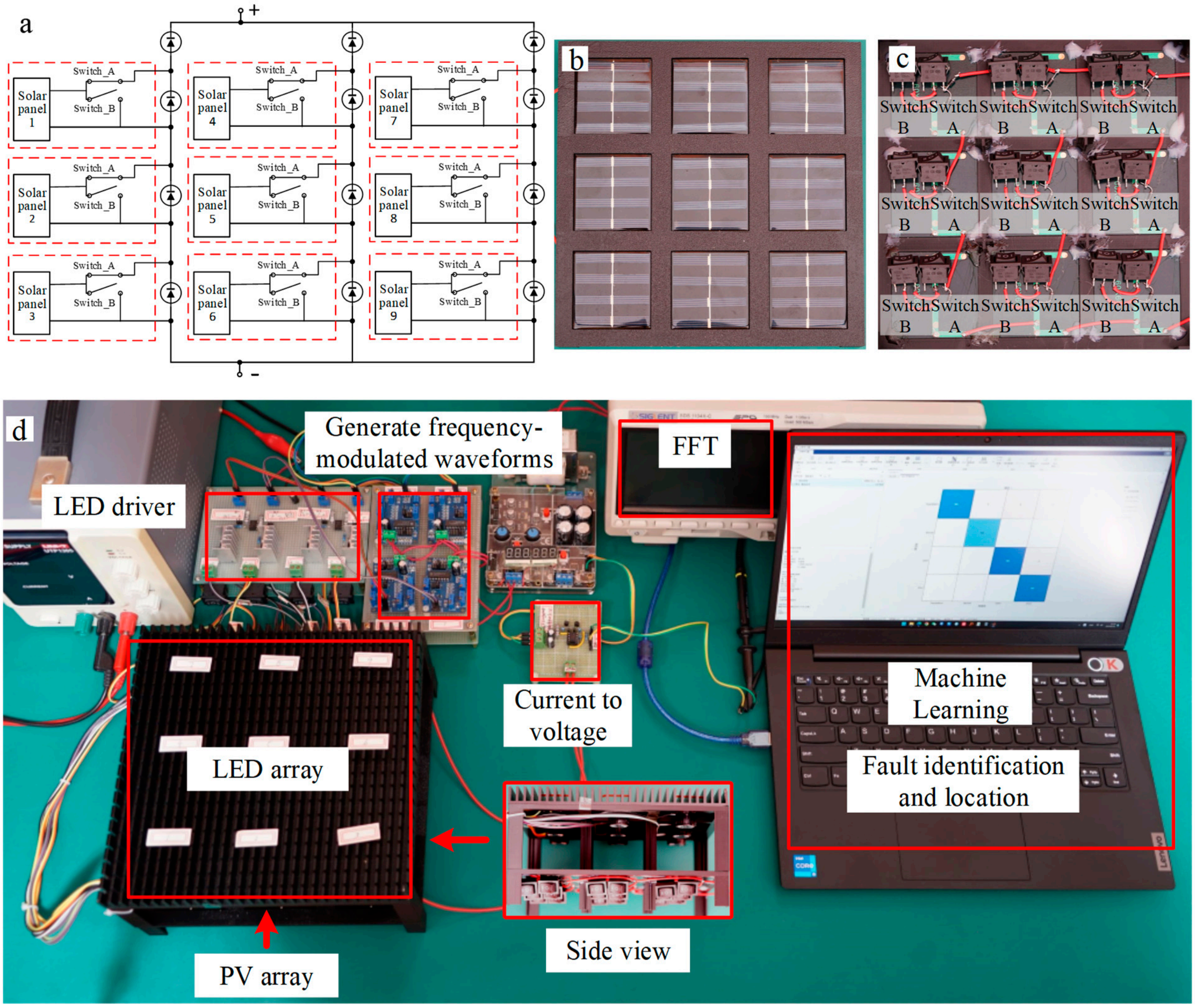

Supplementary Figure S6. The 3×3 series-parallel photovoltaic array and experimental setup. (a) Circuit schematic diagram. (b) Photograph (front view). (c) Photograph (back view). (d) Photograph (Experimental setup).

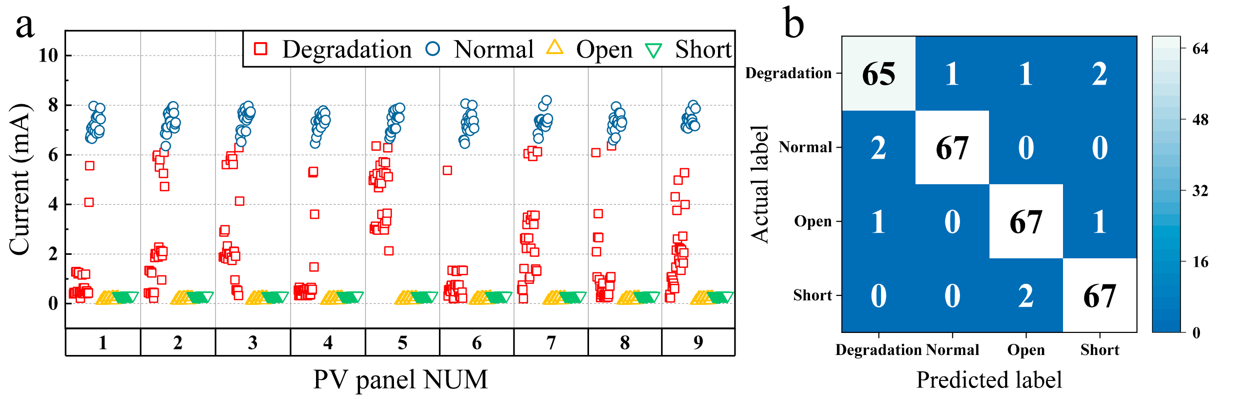

Supplementary Figure S7. Data set and experimental results of a 3×3 series-parallel photovoltaic array. (a) Distribution of the data set. (b) Testing confusion matrix (Neural Network).

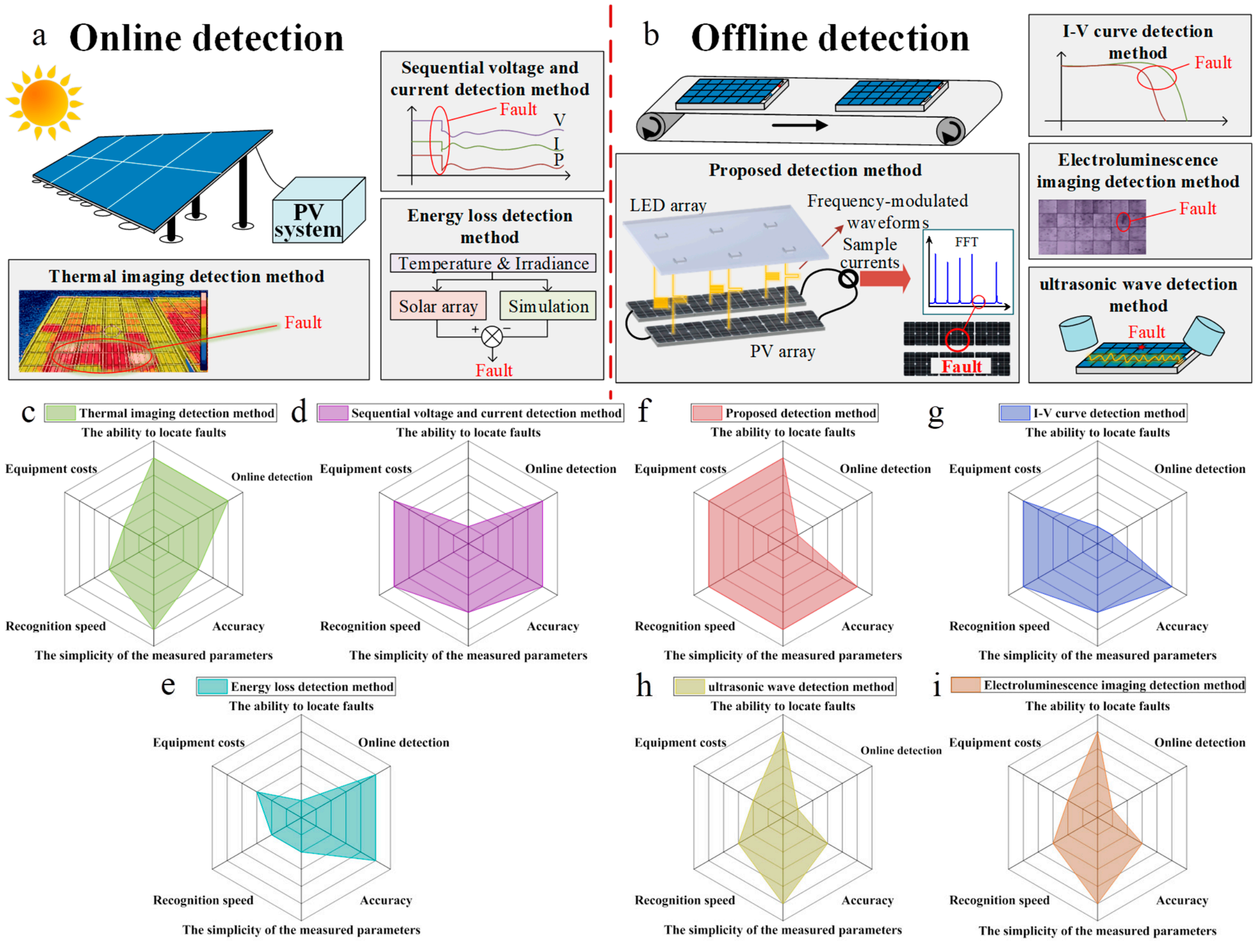

Supplementary Figure S8. The application and comparison of online and offline fault detection methods for photovoltaic arrays. (a) The application of online fault detection methods. (b) The application of offline fault detection methods. (c) The characteristics of the thermal imaging detection method. (d) The characteristics of the sequential voltage and current detection method. (e) The characteristics of the energy loss detection method. (f) The characteristics of the proposed detection method. (g) The characteristics of the I-V curve detection method. (h) The characteristics of the ultrasonic wave detection method. (i) The characteristics of the electroluminescence imaging detection method.

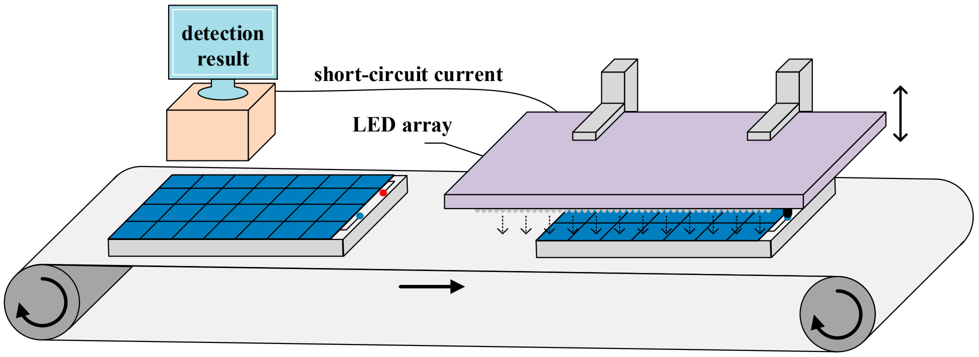

Supplementary Figure S9. Using the method proposed in this paper to perform automated fault detection and localization on photovoltaic arrays during the production process.

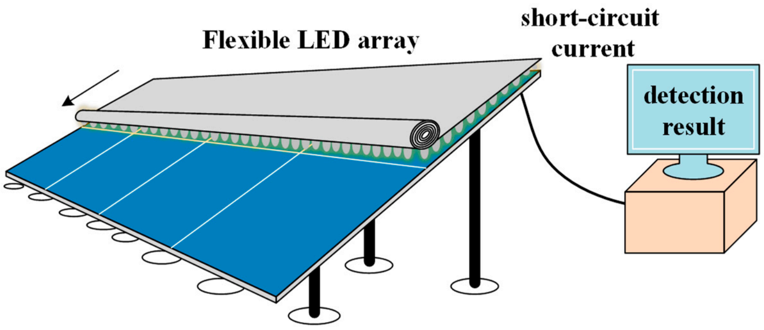

Supplementary Figure S10. Using the method proposed in this paper to perform automated fault detection and localization on outdoor-installed photovoltaic arrays during the maintenance process.

Supplementary Tables

Supplementary Table S1. The Cost Estimates for Different Diagnostic Equipment with Fault Location Capability

The Cost Estimates for Different Diagnostic Equipment with Fault Location Capability (The diagnostic area is 1 square meter)

Type of Equipment	Core Part Name	Quantity	Core Part Cost Estimates (USD)	Equipment Cost Estimate (USD)
This work	LED	20	0.5	132
	LED driver chip	20	1	
	Current detection chip	1	2	
	Neural network computing unit	1	100	
Electroluminescence	High-resolution infrared camera	1	800	1800
	Image recognition computing unit	1	1000	
Thermal imaging	High-resolution infrared camera	1	800	1800
	Image recognition computing unit	1	1000	
Ultrasonic	Ultrasonic generator	1	30	610
	Ultrasonic sensor	1	30	
	Three-axis motion platform	1	450	
	Digital signal processor	1	100	

Supplementary Table S2. The Parameters of the Experimental Platform.

The Parameters of the Experimental Platform

Type of Equipment	Parameters
DC Power Supply	Manufactor: UNI-T<br>Product model: UTP1305<br>Output Voltage: 0-32 V (Set to 12 V)<br>Output Current: 0-5 A (Set to 5 A)
Oscilloscope	Manufactor: SIGLENT<br>Product model: SDS1104X-C<br>Bandwidth: 100 MHz<br>Sampling Rate: 1 GSa/s<br>Memory Depth: 14 Mpts/CH
LED Array	Color: Full Spectrum White Light<br>Power: 5 W<br>Transmission Angle: 30 °
LED Driver	Transconductance: 1 S<br>Output Current: 0-1 A<br>Frequency Range: 0-200 kHz
Signal Generation Module	Output Waveforms: Square, Triangle, Sine<br>Frequency Range: 10 Hz-450 kHz<br>Output Voltage Amplitude:0-12 V (Set to 1 V)<br>Temperature Drift: <50 ppm/°C
Current to Voltage Module	Transresistance: 10 Ω<br>Input Current: 0-1 A<br>Frequency Range: 0-200 kHz
Photovoltaic Array	Number of Series Connections: 2<br>Number of Parallel Connections: 2<br>Output Voltage: 4 V<br>Output Current: 200 mA

Supplementary Notes

Supplementary Note S1: The analysis of the impact of simplifying (1) with (2) on the method presented in this paper.

The equivalent series resistance (R_s) is typically approximately zero, while the equivalent parallel resistance (R_{sh}) is much larger than R_s. For example, in reference [1], R_s equals 0.1 Ω, and R_{sh} equals 644.3 Ω. The method presented in this paper requires setting the photovoltaic array to a short-circuit state. Figure S2(a) illustrates the I-V curve of the photovoltaic panel, with the ideal short-circuit state indicated by the arrow. R_s affects the voltage when the photovoltaic panel is short-circuited, causing the corresponding position on the I-V curve to shift to the right to the actual short-circuit state, as shown in Figure S2(a). Since the slope at this point on the I-V curve is approximately zero [2], the change in current can be neglected. In the short-circuit state, R_{sh} and R_s are in parallel, as shown in Figure S2(b). Since R_{sh} is much larger than R_s, the impact of the current division on the measurement can be safely disregarded. Therefore, to simplify the analysis, (2) is used to simplify (1).

To further demonstrate that the simplification does not impact the diagnostic effectiveness, we conducted simulations using parameters from reference [1]. The simulation circuit is shown in Figure S2(b). Switch 1 and switch 2 control the connection of R_{sh} and R_s. When R_{sh} and R_s are not connected, the measured short-circuit current is 10 mA. When R_{sh} and R_s are connected, the measured short-circuit current is 9.998 mA, representing a 0.2‰ decrease of current compared to the case where R_{sh} and R_s are connected. The simulation results indicate a minimal influence of R_{sh} and R_s on the short-circuit current measurement in the photovoltaic panel. Therefore, simplifying (1) with (2) does not render the method presented in this paper ineffective.

Supplementary Note S2: A demo system for fault diagnosis of photovoltaic panel arrays based on the method proposed in this paper.

The system comprises an array of LEDs, an LED driver, an I-V conversion circuit, an ARM system, and host software, with a fault diagnosis model based on neural networks deployed on ARM. The host software is used to display the photovoltaic current magnitude of each photovoltaic panel as well as its fault status. This device can accurately diagnose and locate each photovoltaic panel's four states (normal, short-circuit, open-circuit, degradation) in the array, unaffected by external light sources, as shown in Figure S3 and Supplementary Video-1.

Under normal conditions, the system measures the normalized photovoltaic current of each photovoltaic panel as 100%, accurately identifying all four photovoltaic panels as usual. Maintaining the states of other photovoltaic panels unchanged, sequentially setting photovoltaic panel 2 to open-circuit, short-circuit, and degradation fault states, the system rapidly (within 1 s) and accurately identifies the fault states of photovoltaic panel 2. In contrast, the states of other panels remain unaffected. Similarly, maintaining the states of other photovoltaic panels unchanged, setting photovoltaic panel 1 to open-circuit and short-circuit fault states successively, the system rapidly (within 1 s) and accurately identifies the fault states of photovoltaic panel 1, while the states of other panels remain unaffected. Keeping the states of photovoltaic panel 1 and 2 unchanged, setting photovoltaic panel 3 to an open-circuit fault state and photovoltaic panel 4 to a degradation fault state, the system rapidly (within 1 s) and accurately identifies the status of each photovoltaic panel. Finally, illuminating the surface of the photovoltaic panel array with external light does not affect the measurement results. This demonstrates the high accuracy, fast speed, low cost, minimal measurement requirements, and fault localization capabilities of the method proposed in this paper.

Supplementary Note S3: A more complex series-parallel photovoltaic array structure is used to verify the feasibility and performance of the method in this paper.

We constructed a 3×3 series-parallel photovoltaic array to demonstrate that the method proposed in this paper can be applied to a more complex series-parallel structured photovoltaic array. The schematic diagram is shown in Figure S6(a). Photographs are shown in Figure S6(b) and (c). Using the experimental platform designed in this study, we collected 920 data sets from the photovoltaic array under four states: normal, short-circuit fault, open-circuit fault, and degradation fault, as shown in Figure S6(d). The data distribution is shown in Figure S7(a).

We used 70% of the data set for training and the remaining 30% for testing. We retrained the neural network model using this data set. We evaluated its performance, as shown in Figure S7(b). The experimental results show that the method proposed in this paper achieved a test accuracy of 96.4% in fault identification and localization for the 3×3 structured photovoltaic array. This demonstrates the applicability of the method proposed in this paper to complex photovoltaic array structures.

Supplementary Note S4: A detailed comparison and application analysis of the proposed method and existing methods.

Photovoltaic array fault detection methods are divided into online type and offline type. The online method is primarily used to monitor the states of photovoltaic arrays during their operation. In contrast, the offline method is mainly employed for testing photovoltaic arrays during their production and maintenance [3-5]. Both online and offline fault diagnosis techniques for photovoltaic arrays are very important. The photovoltaic array fault diagnosis method proposed in this paper is offline and mainly used for detection during production and maintenance. Currently, the main methods used for offline diagnosis of photovoltaic arrays during production and maintenance include 1. electroluminescence imaging detection method, 2. ultrasonic wave detection method, and 3. I-V curve detection method. Electroluminescence imaging and ultrasonic wave detection methods can identify fault types and locate fault positions. However, these methods indirectly assess the state of photovoltaic cells by measuring their electroluminescence imaging or mechanical properties, so they cannot directly obtain information on the photoelectric conversion performance. As a result, their detection accuracy is low, and the equipment costs are high. The I-V curve detection method offers higher detection accuracy but cannot pinpoint the location of the fault.

Online photovoltaic array fault diagnosis methods include 1. energy loss detection method, 2. sequential voltage and current detection method, and 3. thermal imaging detection method. While the energy loss detection method and sequential voltage and current detection method offer high detection accuracy, they cannot pinpoint the fault location. The thermal imaging detection method can both identify the type of fault and locate its position. However, this method indirectly assesses the state of photovoltaic cells by measuring their thermal imaging and cannot directly obtain information on photoelectric conversion performance. As a result, its detection accuracy is low. Although the method proposed in this paper is not an online method, it has advantages in terms of detection accuracy, localization capability, equipment cost, detection speed, and the simplicity of the required measurement parameters. The application and comparison of online and offline fault detection methods are shown in Figure S8.

The diagnostic methods proposed in this paper can be applied in real-world scenarios as follows.

The method proposed in this paper can be applied in the automated production and maintenance of photovoltaic arrays using the following device, as shown in Figure S9. Compared to 1. electroluminescence imaging detection method, 2. ultrasonic wave detection method, and 3. I-V curve detection method, it offers high-precision detection performance and localization capabilities.

The working process of the device is as follows: The device transports the photovoltaic cells beneath the robotic arm that installs the LED array. The robotic arm places the LED array above the photovoltaic cell to be tested. While connecting to the output electrodes of the photovoltaic cell, the device also blocks external ambient light. Finally, the device performs fault diagnosis and localization using the method proposed in this paper, as shown in Figure S9.

The method proposed in this paper can be applied using the following device for testing during the maintenance process of outdoor photovoltaic arrays, as shown in Figure S10. Compared to 1. thermal imaging detection method, 2. sequential voltage and current detection method, and 3. energy loss detection method, the method in this paper offers higher detection accuracy and the ability to locate faults.

The working process of the device is as follows: When a fault occurs in an outdoor photovoltaic array, the maintenance worker unfolds the flexible LED array and places it over the faulty array. The LED array blocks external ambient light. The output electrodes of the photovoltaic array are then connected to the device for fault diagnosis and localization. Finally, based on the diagnostic results, the maintenance worker replaces the damaged components, as shown in Figure S10.

References

[1] Z. Chen, Y. Chen, L. Wu, S. Cheng, and P. Lin, Deep residual network based fault detection and diagnosis of photovoltaic arrays using current-voltage curves and ambient conditions, Energy Conv. Manag. 198 (2019)

[2] J.-M. Huang, R.-J. Wai, and W. Gao, Newly-Designed Fault Diagnostic Method for Solar Photovoltaic Generation System Based on IV-Curve Measurement, IEEE Access, Article. 7 (2019) 70919-70932.

[3] K. AbdulMawjood, S. S. Refaat, W. G. Morsi, and Ieee, Detection and Prediction of Faults in Photovoltaic Arrays: A Review, in Proc. - IEEE Int. Conf. Compat., Power Electron. Power Eng., CPE-POWERENG, Doha, QATAR, Apr 10-12 2018, 1-8.

[4] B. Long-Dong, Y. K. Wu, and M. H. Pham, Fault identification and diagnosis methods for photovoltaic system: A review, in Int. Conf. Appl. Syst. Innov., ICASI, 2021, 126-129.

[5] S. R. Madeti and S. N. Singh, A comprehensive study on different types of faults and detection techniques for solar photovoltaic system, Sol. Energy. 158 (2017) 161-185.
